# ﻿Complete distribution of the genus *Laevilitorina* (Littorinimorpha, Littorinidae) in the Southern Hemisphere: remarks and natural history

**DOI:** 10.3897/zookeys.1127.91310

**Published:** 2022-11-02

**Authors:** Sebastián Rosenfeld, Claudia S. Maturana, Hamish G. Spencer, Peter Convey, Thomas Saucède, Paul Brickle, Francisco Bahamonde, Quentin Jossart, Elie Poulin, Claudio Gonzalez-Wevar

**Affiliations:** 1 Laboratorio de Ecosistemas Marinos Antárticos y Subantárticos, Universidad de Magallanes, Punta Arenas, Chile; 2 Millennium Institute Biodiversity of Antarctic and Subantarctic Ecosystems (BASE), Las Palmeras 3425, Santiago, Chile; 3 Cape Horn International Center (CHIC), Puerto Williams, Chile; 4 Centro de Investigación Gaia‑Antártica, Universidad de Magallanes, Avenida Bulnes 01855, Punta Arenas, Chile; 5 Institute of Ecology and Biodiversity (IEB), Las Palmeras 3425, Santiago, Chile; 6 Department of Zoology, University of Otago, Dunedin, New Zealand; 7 British Antarctic Survey (BAS), Cambridge, UK; 8 Department of Zoology, University of Johannesburg, Johannesburg, South Africa; 9 Biogéosciences, UMR 6282 CNRS, Université Bourgogne Franche-Comté, 6, boulevard Gabriel, 21000, Dijon, France; 10 South Atlantic Environmental Research Institute, Ross Road, Stanley, Falkland Islands, UK; 11 School of Biological Sciences (Zoology), University of Aberdeen, Aberdeen, UK; 12 Marine Biology, Université Libre de Bruxelles (ULB), Brussels, Belgium; 13 Centro FONDAP IDEAL, Instituto de Ciencias Marinas y Limnológicas (ICML) Facultad de Ciencias, Universidad Austral de Chile, Casilla 567, Valdivia, Chile

**Keywords:** Antarctic, endemism, Laevilitorininae, sub-Antarctic

## Abstract

Littorinid snails are present in most coastal areas globally, playing a significant role in the ecology of intertidal communities. *Laevilitorina* is a marine gastropod genus distributed exclusively in the Southern Hemisphere, with 21 species reported from South America, the sub-Antarctic islands, Antarctica, New Zealand, Australia and Tasmania. Here, an updated database of 21 species generated from a combination of sources is presented: 1) new field sampling data; 2) published records; 3) the Global Biodiversity Information Facility (GBIF) and The Atlas of Living Australia (ALA), to provide a comprehensive description of the known geographic distribution of the genus and detailed occurrences for each of the 21 species. The database includes 813 records (occurrences), 53 from field sampling, 174 from the literature, 128 from GBIF, and 458 from ALA. West Antarctica had the highest species richness (8 species), followed by sub-Antarctic islands of New Zealand (4 species) and the south-east shelf of Australia (4 species). The provinces of Magellan, New Zealand South Island, and sub-Antarctic Islands of the Indian Ocean include two species each. This study specifically highlights reports of *L.pygmaea* and *L.venusta*, species that have been almost unrecorded since their description. Recent advances in molecular studies of *L.caliginosa* showed that this species does not correspond to a widely distributed taxon, but to multiple divergent lineages distributed throughout the Southern Ocean. Ongoing molecular and taxonomic studies are necessary for a better understanding of the diversity and biogeography of this genus.

## ﻿Introduction

One of the most common challenges facing studies or the construction of inventories of biodiversity is the absence of detailed information on the distribution of taxa throughout the different geographical regions of the planet. Furthermore, species distribution data are usually scattered across different sources of information such as taxonomic reviews, species lists, reports and natural history collections (Beck et al. 2013). Therefore, it is important to merge these different sources into robust and freely accessible biodiversity databases. The Global Biodiversity Information Facility (GBIF) project has enabled the creation of a platform where museums, herbaria and researchers can publish their databases and make them freely available for use (Flemons et al. 2007). However, despite increasing the international effort devoted to the digitisation of specimen catalogues in museums and other repositories, even today only a small proportion of global records are estimated to have been made available online through the efforts of the GBIF and other platforms like the Ocean Biodiversity Information System (OBIS) (Ariño 2010; Maturana et al. 2019; OBIS 2022).

The family Littorinidae represents one of the most conspicuous and abundant components of intertidal communities that inhabit rocky shores across the world’s coasts (Reid 1989). Being such a widespread and accessible group, they have been amongst the most intensively studied marine molluscs (Reid 2007; Reid and Williams 2012; González-Wevar et al. 2022). They play a significant role in the ecology of intertidal communities and have been widely used as models in microevolutionary studies of natural selection and genetic differentiation (Williams et al. 2003; Kess et al. 2018; Estevez et al. 2021; Bosso et al. 2022). In addition, with the advance of molecular tools, the systematics and taxonomy of the family have been updated (Reid and Williams 2004) to give a more accurate classification of species and description of their distributions. Members of the group are present in both hemispheres (Reid 1989; Williams et al. 2003). In the Southern Hemisphere, tropical and temperate species have received most research attention (e.g., Williams et al. 2003; Reid and Williams 2004). As a consequence, while some littorinids are known from southern South America and the Southern Ocean (SO), no recent taxonomic examinations are available and occurrence information remains scarce as and dispersed (Reid 1989).

*Laevilitorina* Pfeffer, 1886 is the most widely distributed genus of marine gastropods present at high latitudes in the Southern Hemisphere (Reid 1989). Its known distribution range includes South America, New Zealand, Australia, Tasmania, and Antarctic (West and East parts), and many peri-(sub)Antarctic islands (South Shetland Islands, South Orkney Islands, Falkland/Malvinas Islands, South Georgia, Crozet, Kerguelen, Heard, Macquarie, Campbell, Auckland, and Antipodes Island). The genus *Laevilitorina* Pfeffer, 1886 is characterised by a thick, generally smooth shell, a non-planktotrophic protoconch and a generally paucispiral operculum (Reid 1989; Warén and Hain 1996). At present, 21 species of *Laevilitorina* are taxonomically accepted (MolluscaBase 2022).

The present study documents the state of knowledge of the genus and provides an updated database, using a combination of recent sampling data, published records available in the literature, and available information from GBIF and other repositories. The objectives of the study are: i) to report new records of *Laevilitorina* species present in Antarctic and sub-Antarctic environments and ii) to evaluate the distribution and richness of *Laevilitorina* species throughout the Southern Hemisphere, using an updated database. The updated database will serve as a basis for future comprehensive systematic research on the genus, including the application of molecular phylogenetic approaches to help infer its regional evolutionary history.

## ﻿Materials and methods

### ﻿Construction of the database

*Laevilitorina* records across the Southern Hemisphere were compiled from four main sources: 1) field sampling data; 2) published literature; 3) data already present in GBIF and 4) the data present in the repository of the Atlas of Living Australia (ALA) (Belbin 2011). Duplicate records were removed to construct a unified database. In addition, the records available in GBIF and ALA were used to describe the distribution range of each species. To ensure the quality of the occurrence data, dubious records were excluded from the geospatial analysis. The criterion used to determine dubious records was records of species in geographic areas outside the distribution range described in the original descriptions and taxonomic revisions.

Twelve marine biogeographical provinces in the Southern Hemisphere were considered for the purpose of our geospatial analyses, including the Magellan province (southern South America and Falkland / Malvinas Islands), West Antarctic, East Antarctica, Indian Ocean sub-Antarctic islands (Prince Edward Islands, Crozet Island, Kerguelen and Heard Islands), Macquarie Island, New Zealand sub-Antarctic islands, Southern New Zealand, Northern New Zealand, South-east Australian Shelf, South-west Australian Shelf, West Central Australian Shelf and East Central Australian Shelf, as defined in Spalding et al. (2007) and Koubbi et al. (2014). All spatial analyses were carried out on the unified database.

#### Recent sampling data

New material was collected from multiple locations in southern South America between the Strait of Magellan (53°36'S, 70°55'W) and the Diego Ramirez archipelago (56°31.345'S, 68°43.622'W). In the Falkland/Malvinas Islands, specimens were collected from the intertidal zone of Hooker Point (51°42'S, 57°46'W). New Antarctic material was collected from the South Shetland Islands, Doumer Island, Palmer Land, and Avian Island under the framework of Antarctic Scientific Expeditions (**ECA**) 49, 53, 54 and 58 of the Chilean Antarctic Institute (**INACH**). Samples from the South Orkney Islands and South Georgia were obtained during BBritish Antarctic Survey (**BAS**) and SAERI expeditions (2016–2017, 2017–2018 and 2021). Samples from Kerguelen and Crozet archipelagos were obtained through the PROTEKER project under the framework of the French Polar Institute Paul Emile Victor (**IPEV**) summer campaign 2017.

#### Sample collection

Samples were collected using two methods: 1) manual collection in the intertidal zone, with littorinids being sampled individually, and 2) SCUBA diving between 1 and 15 m depth, where substrates (e.g. sediments, macroalgae) were collected. Rock substrates were subsequently scraped to ensure that all species and specimens were collected. Each macroalga sample was placed in a plastic bag. After collection, specimens were kept alive and transported onboard or to the research station. Each sample was then gently agitated to detach the associated fauna. All *Laevilitorina* samples were immediately preserved in ethanol (95%) to be transported to the laboratory. Geographic coordinates were recorded using GPS for each sample location.

#### Taxonomic identification

Morphological observations were performed under an OLYMPUS stereomicroscope CX31. The following morphological measurements were taken, following Reid (2007): shell height (H), the maximum dimension parallel to the axis of coiling; shell breadth (B), the maximum dimension perpendicular to H; length of the aperture (LA), the greatest length from the junction of the outer lip with the penultimate whorl to the anterior lip. For determination to species level, each individual was identified following the taxonomic studies of Martens and Pfeffer (1886), Suter (1913), Powell (1951, 1955), Dell (1964), Arnaud and Bandel (1976), Warén and Hain (1996) and Zelaya (2005).

#### Published literature

To ensure maximum coverage of the generated dataset, information was gathered from all available scientific publications that have sampled or reviewed *Laevilitorina* species throughout the genus’ distribution, from the description of the first species (Gould 1849) to the present. These records and their respective geographical positions were entered into a spreadsheet following the Darwin Core Standard structured procedure (Wieczorek et al. 2012). Taxonomy used in these publications was updated following the most recent systematic revision (Reid 1989; Warén and Hain 1996; Engl 2012; Bouchet et al. 2017; MolluscaBase 2022). We did not follow González-Wevar et al. (2022) for species names and databases, mainly because the lineages that would correspond to new species have not yet been formally described. However, the implications of these results for the taxonomy and biogeography of *Laevilitorina* are discussed (González-Wevar et al. 2022).

#### Digital database GBIF and ALA

All georeferenced records of the genus *Laevilitorina* were retrieved from the GBIF and ALA database on 12 September 2022 (Rosenfeld et al. 2022). The point-radius method was used for georeferencing records lacking precise geographic location (coordinates), by identifying locality description included in the relevant metadata of the reported collection. This method considers the precision, datum and specificity of the locality description to determine the coordinates (Wieczorek et al. 2004; Wieczorek and Wieczorek 2021). The species list was updated to exclude erroneous or suspect records, rule out possible synonymy and follow current taxonomy.

## ﻿Results

### ﻿Database summary

The complete database (https://www.gbif.org/dataset/cd023c5e-8729-41b2-b9df-1419289c0e40) includes 813 records. Most records (458) were obtained from the ALA repository, followed by literature (174) obtained from 63 reviewed articles, GBIF (128), and new sampling records (53).

#### Dubious records

*Laevilitorinaantarctica* (Smith, 1902), originally described from Cape Adare in the Ross Sea, is also reported in GBIF from Macquarie Island (https://www.gbif.org/es/occurrence/search?taxon_key=9810991). However, this species has historically been reported primarily from the biogeographic provinces of East Antarctica and West Antarctica (Arnaud and Bandel 1976; Dell 1990). Therefore, the presence of *L.antarctica* on Macquarie Island requires confirmation and was not included in our database.

#### New record

This study includes the first record of the species *Laevilitorinadelli* Powell, 1955, in GBIF database, previously described by Powell (1955) from the South Island of New Zealand and Antipodes Island.

#### Morphological identification

**Figure 1.**
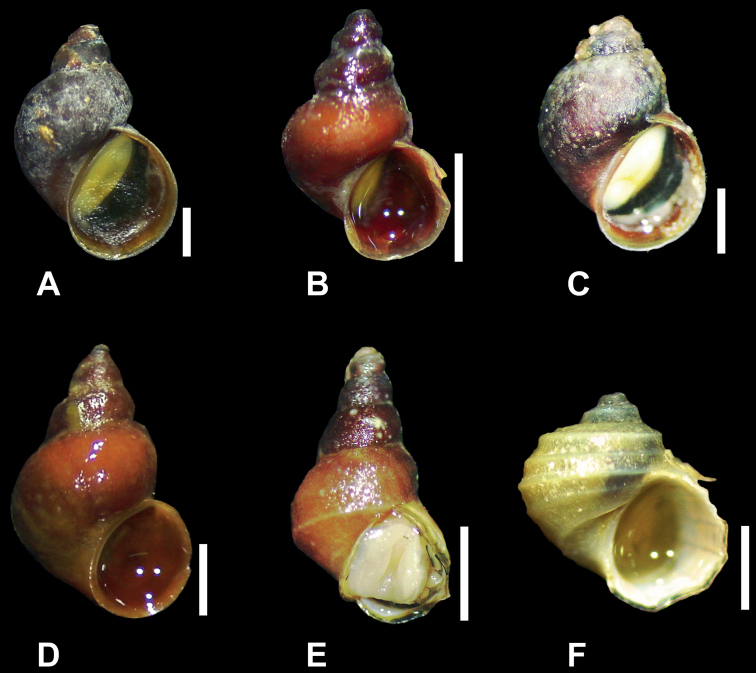
**A***Laevilitorinacaliginosa* (4.8 mm) **B***Laevilitorinapygmaea* (2.5 mm) **C***Laevilitorinavenusta* (3.7 mm) **D***Laevilitorinaclaviformis* (3.9 mm) **E***Laevilitorinaumbilicata* (2.8 mm) **F***Laevilitorinawandelensis* (2.7 mm). Scale bars: 1 mm. Photographs by Sebastián Rosenfeld.

All newly collected *Laevilitorina* specimens identified in this study showed morphological characteristics corresponding to those described in the literature (Fig. 1a–f). The specimens of *L.pygmaea* Pfeffer, 1886 and *L.venusta* Pfeffer, 1886 identified from South Georgia are consistent with the morphological characteristics described by Martens and Pfeffer (1886) for these species (Fig. 1b, c). Individuals of *L.pygmaea* had a high spire, reddish-brown periostracum, with five convex whorls. The last whorl was 50% of the total height of the spire and the aperture was ~ 59% of the length of the last whorl (Fig. 1b). *L.venusta* individuals were between 3.7 and 5.6 mm in height, with a short spire, and 4.5 convex whorls. The aperture was wide, occupying a little more than half of the total height of the shell (54%); the columellar callus was sharp, white and expanded towards the umbilicus, all characteristics again consistent with Martens and Pfeffer (1886) (Fig. 1c).

#### Species richness

**Figure 2.**
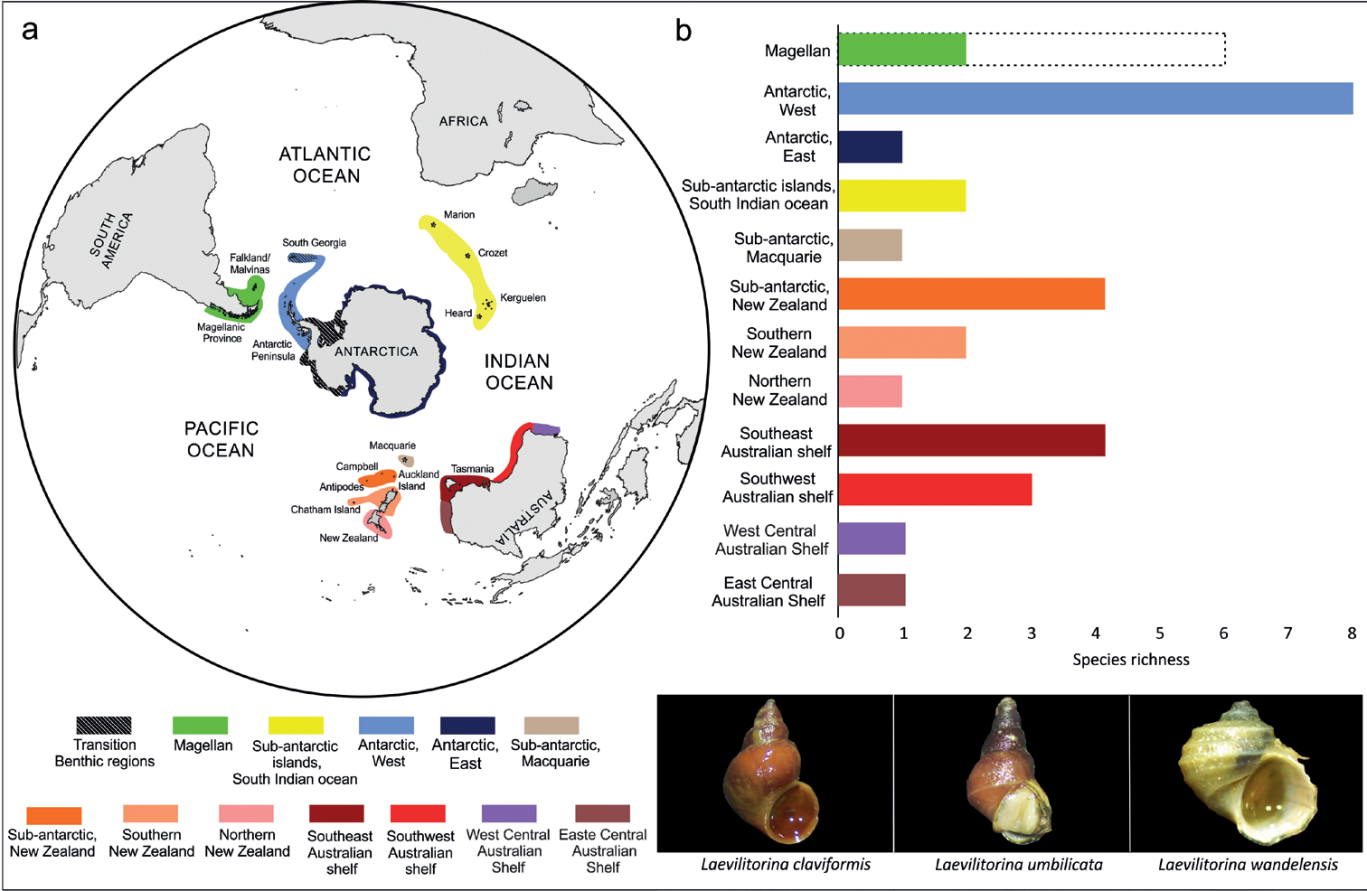
**a** Delimitation of Antarctic and Southern Ocean marine biogeographic provinces according to Spalding et al. (2007) and Koubbi et al. (2014)**b** Species richness of *Laevilitorina* in each of the biogeographical provinces. The dotted lines in the Magellan Province show the new richness value based on the revision of González-Wevar et al. (2022).

A total of 21 species of *Laevilitorina* were recorded in the Southern Hemisphere; West Antarctica was the province with the highest species richness (S = 8, Fig. 2a, b), followed by the New Zealand sub-Antarctic islands, the south-east shelf of Australia (S = 4, Fig. 2a, b) and the south-west Australian Shelf (S = 3, Fig. 2a, b). The provinces of Magellan, south New Zealand, and Indian Ocean sub-Antarctic islands had two species each (Fig. 2 a, b) and the remaining provinces had only one species each (Fig. 2 a, b). However, based on the latest molecular study of González-Wevar et al. (2022), there are four new species-level lineages of *Laevilitorina* in the Magellan province where species richness would increase to six taxa (Fig. 2 a, b). The species with the highest number of records was *L.caliginosa* (Gould, 1849) (158). Most of these records came from the Magellan province (79), of which nine were from the Falkland/Malvinas Islands.

**Figure 3.**
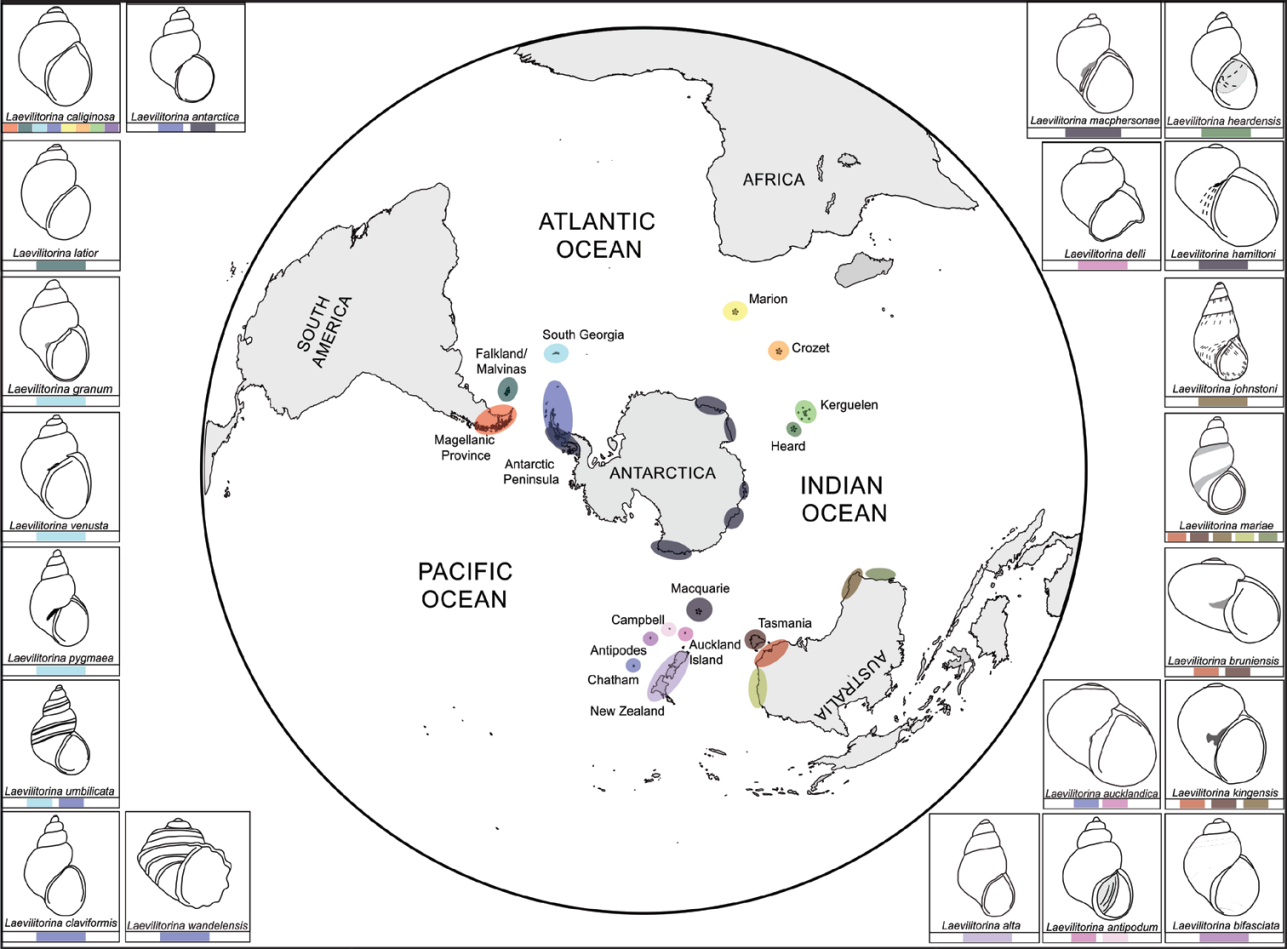
The distributions of the 21 different *Laevilitorina* species in the Southern Hemisphere. The colours below each panel indicate the geographic distribution of each species. Drawings of each species were made from holotypes or from illustrations made in published revisions (Gould 1849; Martens and Pfeffer 1886; Smith 1902; Lamy 1906; Suter 1913; Preston 1916; May 1924; Powell 1933; Powell 1940; Cotton 1945; Powell 1955; Dell 1964).

Within the West Antarctic province eight species were reported, of which *L.venusta* and *Laevilitorinagranum* Pfeffer, 1886 were recorded exclusively from South Georgia (Fig. 3), while *L.wandelensis* (Lamy, 1906) and *L.antarctica* were recorded exclusively from Antarctic provinces, without no records from South Georgia (Fig. 3).

Only two species were recorded from the main New Zealand islands, *L.alta* (Powell, 1940) from North Island and *L.delli* from South Island. Three species were reported from Campbell, Antipodes and Auckland Islands, *L.aucklandica* (Powell, 1930), *L.bifasciata* Suter, 1913 and *L.antipodum* (Filhol 1880), none of which were shared with the North and South Islands of New Zealand (Fig. 3). In Australia, four species, *L.johnstoni* (Cotton, 1945), *L.mariae* (Tenison Woods, 1876), *L.bruniensis* (Beddome, 1883), and *L.kingensis* (May, 1924), were recorded from mainland Australia. *L.johnstoni* would be the only species restricted to mainland Australia, while *L.kingensis*, *L.mariae*, and *L.bruniensis* are also present in Tasmania (Fig. 3).

Based on our new sampling data only, we identified and reported seven *Laevilitorina* species in the Magellanic Province (*L.caliginosa*), Falkland/Malvinas Islands (*L.caliginosa*, *L.latior*), South Georgia (*L.caliginosa*, *L.pygmaea*, *L.venusta*; Fig. 1b, c), Kerguelen and Crozet Islands (*L.caliginosa*), South Orkney Islands (Signy Island) (*L.caliginosa*), and Antarctic Peninsula (*L.caliginosa*, *L.claviformis*, *L.umbilicata*, *L.wandelensis*; Fig. 1d–f), adding 43 new records to the previously available data. These new records are generally consistent with the existing literature and GBIF data, with the exceptions of (i) new records of *L.caliginosa* on Horn and Diego Ramirez Islands, (ii) *L.umbilicata* on Avian Island, and (iii) *L.caliginosa* on Lagotellerie Island, the latter two being the southernmost records of both species.

## ﻿Discussion

The increasing application of integrated taxonomy coupled with new modelling approaches, requires data to be Findable, Accessible, Interoperable, and Reusable in the long term (Wilkinson et al. 2016). There is a need to revise the geographic distribution and taxonomic description of many taxa, as it can provide information about changes in the composition of communities in different environments, particularly in sensitive ecosystems (Maturana et al. 2019). A number of studies have already discussed the importance of making an updated revision of the taxonomic status of several *Laevilitorina* species throughout their distribution (Powell 1960; Reid 1989; Engl 2012).

The compilation and unification of records of *Laevilitorina* in the Southern Hemisphere presented here contributes to improve our knowledge of the diversity and biogeography of the members of the genus in twelve biogeographic provinces of the Southern Hemisphere. However, it is also important to note that, despite the unification and update of records of *Laevilitorina*, this study does not reflect the full systematic and biogeographic complexity of this genus. Distribution data are not currently available for many members of the genus, which have not been reported since their description. For example, among the five species of *Laevilitorina* described from South Georgia, three of them (*L.pygmaea*, *L.venusta*, and *L.granum*) have not been reported since their original description (Castellanos 1989), leaving doubts about the taxonomic validity of these species (Castellanos 1989; Reid 1989; Engl 2012).

In this study, the report of *L.pygmaea* is only the third record of the species, in addition to being the first record from shallow depths thereby extending our knowledge of its bathymetric range. Previously, *L.pygmaea* had been reported between 252 and 310 m depth (Castellanos 1989). Similarly, the record of *L.venusta* is the first report of this species since its description by Martens and Pfeffer (1886). In general, the morphology of new *L.pygmaea* and *L.venusta* specimens corresponded well with the original descriptions. However, in our individuals of *L.pygmaea* the aperture was slightly higher than that described by Martens and Pfeffer (1886). This difference could be due to morphological plasticity within *L.pygmaea*, as it has been reported for other species of the genus (Reid 1989; Engl 2012). In the case of *L.venusta*, our specimens presented characteristics and measurements similar to those described by Martens and Pfeffer (1886), where the length of the opening of our specimens represented ~ 54% of the total height of the shell, the same as the measurements of the holotype of Martens and Pfeffer (1886). The morphology of *L.venusta* is quite similar to that of the widely distributed *L.caliginosa*, a species characterised by wide morphological plasticity throughout its distribution (see Engl 2012; González-Wevar et al. 2022). However, measurements of specimens of *L.caliginosa* from the Falkland/Malvinas Islands and South Georgia show a longer and more expanded aperture than *L.venusta*, occupying between 58 and 67% of the total height of the shell (Castellanos 1989; Zelaya 2005). In this sense, it would be interesting in the future to carry out molecular studies with the species of South Georgia to corroborate the validity of the species described in that site. The recent study by González-Wevar et al. (2022) was able to detect only two lineages of *Laevilitorina* there: i) one that would correspond to *L.caliginosa* and ii) a second lineage that is also distributed in the Antarctic Peninsula and expands its distribution towards sub-Antarctic islands of the Indian Ocean like Marion, Crozet, and Kerguelen. The latter does not resemble any known South Georgian species and probably represents a new species (González-Wevar et al. 2022).

Taxonomic uncertainties within the genus *Laevilitorina* are related both to the morphological plasticity that exists in at least some species (Reid 1989; Engl 2012) and also to practical logistical challenges in accessing species’ type localities and the level of geographical accuracy relating to some records. For example, the type locality of *L.caliginosa* (Gould (1849) is described as “Terra del Fuego”, which covers a large and diverse area and could generate many ambiguities for researchers attempting to collect correctly identified individuals from this locality. Tierra del Fuego is one of the largest islands in southern South America and extends south and east of the Strait of Magellan between the Atlantic and Pacific Oceans. Gould’s description was made using material collected during the “United States Exploring Expedition” carried out between 1838 and 1842 (Gould 1849). Fortunately, in the narrative of this expedition (Wilkes 1845; chapter VI, “Terra del Fuego”) it is specified that the ship was in Orange Bay located in Hoste Island (see Wilkes 1845: 123) when this material was collected. Consequently, the type locality of *L.caliginosa* can be defined as Orange Bay in Hoste Island, and not the coastal area of Tierra del Fuego.

Historically, because of the complexity of obtaining material due to the wide distribution of *Laevilitorina*, taxonomic revisions have been restricted to certain geographic areas (e.g., Powell 1951, 1957; Dell 1964; Arnaud and Bandel 1976; Zelaya 2005; Engl 2012). The most complete review published to date was by Reid (1989), where he analysed material from Antarctica (*L.antarctica*), sub-Antarctic Islands (*L.caliginosa* and *L.hamiltoni*), New Zealand (*L.alta*), and Australia (*L.bruniensis* and *L.mariae*). This represents a very low percentage of the diversity of the entire genus. In addition, some of the described species present morphological similarities, which makes identification more complex (Reid 1989) and therefore caution must be exercised with some historical records. Fortunately, several of the described species have material deposited in museums (e.g., ALA 2022), which would allow a more extensive revision of the group. Therefore, a systematic revision of *Laevilitorina* is currently very relevant to understand better the current status of this genus, its richness and distribution in the Southern Hemisphere.

**Figure 4.**
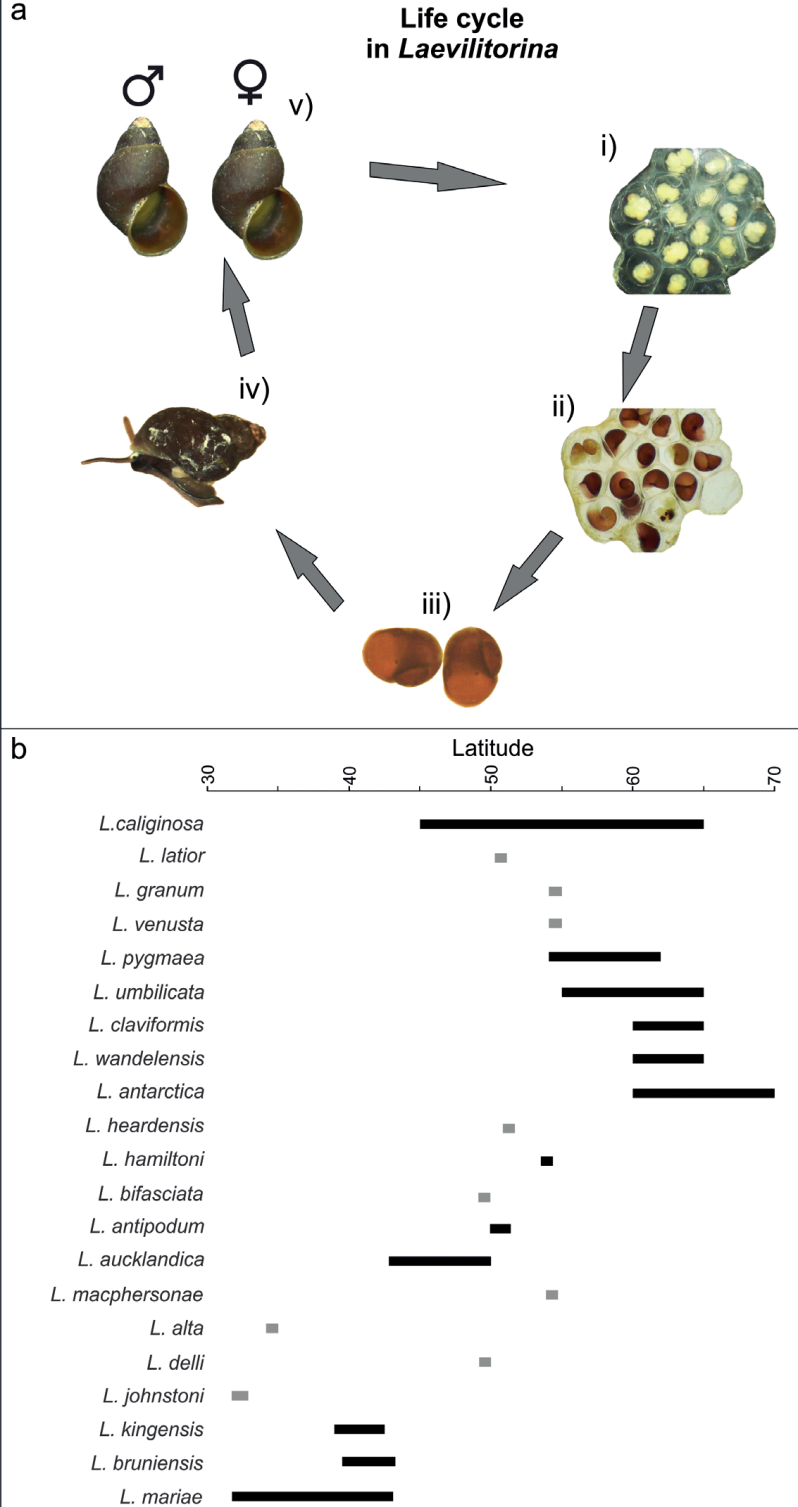
**a** Life cycle of members of the genus *Laevilitorina* without a planktotrophic larval stage, i) general view of the egg mass with early-stage embryos, ii) late-stage embryos, iii) recently hatched juveniles, iv) developing adult, and v) male and female of the genus (photographs S. Rosenfeld) **b** Latitudinal distribution of *Laevilitorina* species in the Southern Hemisphere, grey bars indicate presence in a single geographic area or island.

*Laevilitorina* is one of the most widely distributed genera of marine gastropods at high latitudes in the Southern Hemisphere (Reid 1989; this study). The 21 species of *Laevilitorina* have different distribution patterns (Fig. 3). For example, seven of the 21 *Laevilitorina* species reported in this study have different distribution ranges (*L.caliginosa*, *L.latior*, *L.pygmaea*, *L.venusta*, *L.claviformis*, *L.umbilicata*, *L.wandelensis*) (Fig. 4b). *Laevilitorinalatior* has been reported exclusively from the Falkland/Malvinas Islands (Preston 1912), *L.claviformis* and *L.wandelensis* exclusively from Antarctic Peninsula (Reid 1989; Engl 2012), and *L.venusta* only from South Georgia (Castellanos 1989; Zelaya 2005). *Laevilitorinaumbilicata* and *L.pygmaea* have wider distribution ranges, including both South Georgia and the Antarctic Peninsula (Zelaya 2005; Engl 2012). *Laevilitorinacaliginosa* has by far the widest distribution, being recorded in four Southern Ocean biogeographic provinces (i.e., Magellan, West Antarctica, Indian Ocean sub-Antarctic, and Macquarie Island). Nevertheless, as previously stated, the taxonomy within this taxon is much more complex than previously thought (González-Wevar et al. 2022).

The majority of *Laevilitorina* species inhabit shallow rocky coasts and may be associated with different species of macroalgae (Simpson 1972; Reid 1989; Amsler et al. 2015; Rosenfeld et al. 2017). Another important characteristic of this genus is the absence of pelagic larva: the female deposits egg masses on rocks or macroalgae from which the juvenile subsequently hatches (Picken 1979; Simpson and Harrington 1985) (Fig. 4a). In the literature, this type of benthic protected development is often assumed to be associated with restricted dispersal capability and hence narrow geographic range (Simpson and Harrington 1985; Barroso et al. 2022), a feature of the majority of *Laevilitorina* species (Fig. 4b). On the basis of reproductive strategy, the wide distribution of *L.caliginosa* is paradoxical and exceptional within the genus (Reid 1989; Griffiths and Waller 2016) (Fig. 4b). Some authors (Griffiths and Waller 2016; González-Wevar et al. 2022) have suggested that dispersal associated with dislodged rafts of the seaweed *Durvillaeaantarctica* Hariot, 1882 may have facilitated the species’ wider establishment, since both species co-occur across most of their distribution ranges. However, a recent phylogenetic study of *L.caliginosa* evidenced that this taxon does not correspond to a widely distributed species, but rather to multiple divergent lineages distributed along the SO (González-Wevar et al. 2022). In fact, phylogenetic reconstructions recognised the presence of at least seven *Laevilitorina* lineages within the nominal taxon *L.caliginosa*. Of these, six species are endemic to the Magellan Province and most of them are new to science (González-Wevar et al. 2022). Just one “caliginosa” lineage has a broad distribution that includes the Antarctic Peninsula, South Georgia and sub-Antarctic islands of the Indian Ocean (Marion, Crozet, and Kerguelen islands) (González-Wevar et al. 2022). Hence, the taxonomy of *Laevilitorina* is still unsettled and requires a detailed revision. Previously the Magellan province was considered as a species-poor area for *Laevilitorina*, in fact it represents an area where the genus diversified over the last 30 million years (González-Wevar et al. 2022).

This study shows a detailed review of the records, distribution and richness patterns of the genus *Laevilitorina* throughout its range. However, more research and sampling effort is still needed to “recover” and confirm many of the *Laevilitorina* species that are present throughout the sub-Antarctic Islands. In addition, based on the results of González-Wevar et al. (2022) and this work, we conclude that it is important to continue investigating this genus because: i) the recent discovery of new lineages in the Magellan province highlights the need for a thorough taxonomic revision of *Laevilitorina* species and improved estimate of the genus diversity, and ii) the marked endemism of some species along with differences in species richness across the Southern Hemisphere marine provinces suggest contrasting biogeographical patterns of importance for conservation issues and evolutionary studies. Finally, these differences raise further questions about the underlying processes and mechanisms associated with the evolution of this genus in the Southern Hemisphere.
